# Automated detection of primary soft tissue sarcomas of the extremities using artificial intelligence and ChatGPT

**DOI:** 10.3389/fonc.2026.1674509

**Published:** 2026-03-16

**Authors:** Hendrik Voigtländer, Fabian Schmitz, Dimitrios Strauss, Hans-Ulrich Kauczor, Sebastian Voigtländer, Svea Sauerwein, Sam Sedaghat

**Affiliations:** 1 Department of Diagnostic and Interventional Radiology, University Hospital Heidelberg, Heidelberg, Germany; 2 Carl von Ossietzky Universität Oldenburg, Department of Computer Science, Oldenburg, Germany

**Keywords:** artificial intelligence, ChatGPT, convolutional neural network, MRI, soft-tissue sarcoma

## Abstract

**Objectives:**

Developing effective Convolutional Neural Networks (CNN) for soft tissue sarcoma detection often requires numerous iterations and adjustments, demanding specialized IT (Information Technology) skills. This study aims to use ChatGPT 4 to simplify CNN adaptation, reducing the need for specialized IT skills while enabling efficient exploration of training configurations to enhance diagnostic accuracy.

**Methods:**

This study leveraged a preexisting Artificial Intelligence (AI) model adapted using a preexisting Convolutional Neural Network (CNN). The study involved 54 participants diagnosed with primary soft tissue sarcomas in the extremities and possessing complete Magnetic Resonance Imaging (MRI) datasets. AI adaptations and programming were conducted using TensorFlow and verified with ChatGPT. Model training involved a dataset split of 70% training, 15% validation and 15% test set on patient level split, processed over eight epochs.

**Results:**

The adapted CNN model demonstrated significant improvement across various MRI sequences, achieving high accuracy levels (up to 98.5%) and excellent sensitivity and specificity rates. The model performed robustly in differentiating tumor presence in MR images, with test accuracies as high as 93.9%. The inclusion of a Gradient-weighted Class Activation Mapping (Grad-CAM) heat map and probability scores in the diagnostic outputs further enhanced interpretative capabilities.

**Conclusion:**

This study highlights the potential of AI, particularly CNNs, in the early and accurate detection of soft tissue sarcomas, underscoring the technology’s adaptability across different imaging modalities. The integration of large language models like ChatGPT into the model adaptation process emphasizes the reduced need for specialized IT skills, making advanced diagnostic tools more accessible and potentially improving diagnostic accuracy and patient outcomes in radiology and oncology.

## Introduction

Soft-tissue sarcomas (STS) represent a diverse group of tumors, characterized by their heterogeneity across various histological subtypes and clinical manifestations (1–3). The annual incidence of soft tissue sarcomas ranges from 1.8 to 5.0 per 100,000 individuals, peaking around the age of 60. This incidence is higher in older patients and shown to be stable over the last decades but is expected to rise in an aging society (4–6). These tumors pose significant diagnostic challenges due to their rarity and the often nonspecific nature of their clinical presentation, complicating early and accurate detection which is crucial for effective treatment and improved patient outcomes (7, 8). Ongoing research has focused on identifying imaging features that can reliably predict soft tissue sarcoma grade. Among the most valuable Magnetic Resonance Imaging (MRI)-based features are intratumoral heterogeneity, tumor margins, tumor size, peritumoral enhancement, peritumoral edema, and tumor configuration or shape (9–13). However, the detection of primary soft tissue sarcomas in the extremities presents significant challenges in the field of oncology (1, 14–16). Recently, Artificial Intelligence (AI) has emerged as a promising tool in medical diagnostics, improving the accuracy and efficiency of disease detection and classification (17). AI’s capabilities also extend to aiding in the selection of appropriate therapeutic interventions and supporting clinical decision-making (18). Despite these advancements, the optimal Convolutional Neural Network (CNN) for STS detection has yet to be developed, as its creation often requires significant IT expertise (19).

This paper investigates the adaptation of a preexisting AI model for detecting primary soft-tissue sarcomas in the extremities, using ChatGPT to streamline programming.

## Methods

### Participants

This study was conducted with approval from the Institutional Review Board (IRB) of the participating hospital. The IRB granted a waiver for written informed consent due to the retrospective nature of the study.

### Inclusion and exclusion criteria

Participants were eligible for inclusion if they had a primary diagnosis of soft tissue sarcoma in the extremities and provided a complete MRI dataset. This dataset had to include at least one T1-weighted (T1) and one T2-weighted (T2) image. Participants were excluded if they had received prior treatment for sarcoma, had recurrent disease, had incomplete MRI data, liposarcomas, angiosarcomas, or retroperitoneal sarcomas. A total of 54 patients were enrolled in the study (**Table 1**).

**Table 1:** Clinical information on the 54 patients included in CNN training.

Category	Information
Patient Count	54
Gender	
•Female	55% (n=30)
•Male	45% (n=24)
Mean Age	49.8 +/- 19.8 years
Tumor Type	
•Synovial Sarcoma	24% (n=13)
•Myxofibrosarcoma	14.5% (n=8)
•Other	61.5% (n=33)
Location	
•Thigh	38% (n=21)
•Upper Arm	13% (n=7)
•Other	48% (n=26)
Mean Tumor Size	54.5 +/- 33.4 mm

Patients with liposarcomas, angiosarcomas, and retroperitoneal sarcomas were excluded from the study.

### Dataset preparation

**Figure 1:**
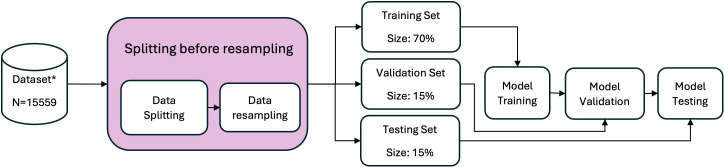
Schematic overview of workflow with the preprocessing and model-development pipeline. The dataset is split at the patient level into training, validation, and test sets (data splitting). Subsequently, resampling is applied within the training data (data resampling). Model development, including hyperparameter tuning, is performed using the training and validation sets, while the test set is used only for final evaluation.

For each examination, 2D slices were exported from available 3D and 2D MRI series in the axial, coronal, and sagittal planes (T1, T1Gd, T2, T2fs) and converted into a unified 2D image format for CNN input. MR images were acquired on multiple scanners and organized into a structured database. Two radiologists (H.V., 4 years of experience; S.S., 10 years of experience) independently labeled each 2D slice as “tumor yes” or “tumor no” after reviewing the complete examination; discrepancies were resolved by consensus re-review. All images were resized to 224 × 224 pixels, and all slices were retained to reflect routine clinical acquisitions, yielding 15,559 images in total (**Table 2**). The dataset was split at the patient level into training (70%), validation (15%), and test (15%) sets (**Figure 1**).

**Table 2:** Overview of the MRI dataset used for CNN-based soft-tissue tumor detection, summarizing the number of examinations and images, the proportion of tumor-containing images, and the distribution of sequences and imaging planes, including the share of non–contrast-enhanced studies.

Category	Information
Numbers of Examinations	54
•Examination without contrast agent	11 (20.4%)
Image Count	15559
Average number of images per examination	273.9
tumor-containing images	5392 (34.7%)
Number of images per MR sequences	
•T2	1293 (8.3%)
•T2 fs	6840 (44.0%)
•T1	3930 (25.3%)
•T1 Gd	3496 (22.4%)
Number of images per imaging plane	
•axial	8801 (56.6%)
•coronal	4390 (28.2%)
•sagittal	2368 (15.2%)

To obtain the “Overall” (multi-modality) result, we used data-level aggregation (pooled training): after the patient-level split, all 2D slices from the available MRI sequences (T1, T1Gd, T2, T2fs) were pooled within each split to form a single multi-modality dataset. We then trained one CNN on the pooled multi-modality training set (“Overall”). In addition, to report modality-specific performance, we trained separate CNNs for each modality using only slices of the respective sequence (T1-only, T1Gd-only, T2-only, T2fs-only), while keeping the same patient-level split strategy. For evaluation, “Overall” metrics were computed on the pooled multi-modality test set, whereas modality-specific metrics were computed on the corresponding modality-specific test subsets.

### Model development and ChatGPT-4–assisted adaptation workflow

**Figure 2:**
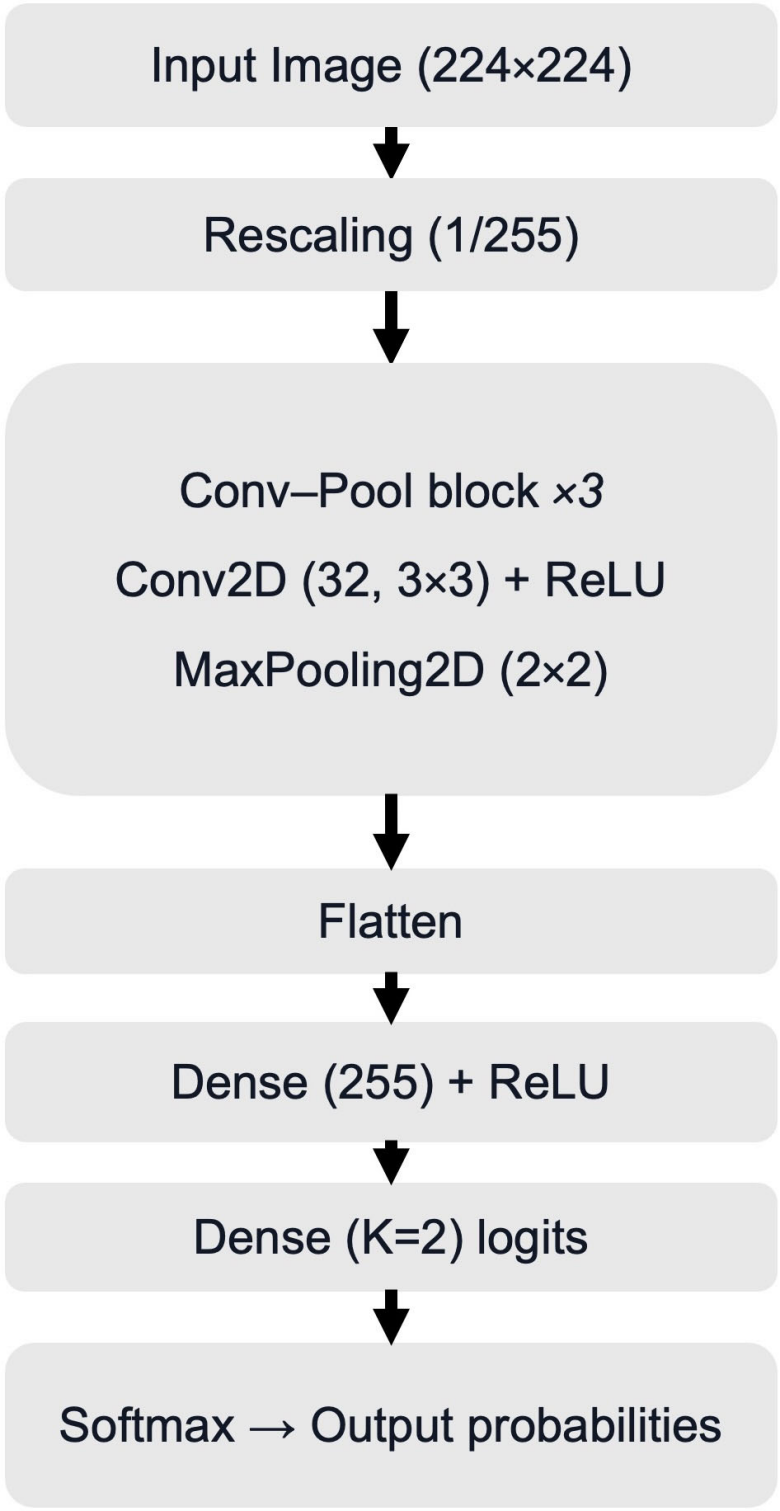
Baseline CNN architecture for binary classification (sarcoma vs. no sarcoma). The diagram shows the processing of preprocessed MRI-derived 2D input images through repeated Conv2D–ReLU–MaxPooling2D blocks, followed by fully connected layers and a 2-class softmax output (K = 2). The same architecture was used across all baseline experiments. CNN, convolutional neural network; Conv2D, two-dimensional convolution; ReLU, rectified linear unit; MaxPooling2D, two-dimensional max pooling; K, number of classes. logits, unnormalized outputs before softmax; softmax, function converting logits to class probabilities.

A baseline Keras Sequential CNN was implemented following the official TensorFlow image-classification (20). The architecture consisted of an input rescaling layer, three convolution–pooling blocks, a flattening step, and two fully connected layers (**Figure 2**). Transfer learning was not used. The CNN was trained from scratch with randomly initialized weights; no pretrained backbone was employed and no layers were frozen (all layers were trainable throughout training).

To adapt the baseline to the present soft-tissue sarcoma dataset, we implemented task-specific adjustments focused on (A) standardized data ingestion and reproducible, patient-level splitting; (B) output configuration and loss definition for the binary label space (K = 2); (C) stable and efficient training via caching, prefetching, and consistent batching; and (D) evaluation outputs and interpretability artifacts beyond accuracy, including confusion matrix–based measures and per-image prediction exports to support systematic error analysis. For binary classification, the network used a two-logit output layer (Dense(2), no activation) trained with Sparse Categorical Cross-Entropy loss (from_logits=True); tumor probability was obtained by applying softmax to the logits.

**Figure 3:**
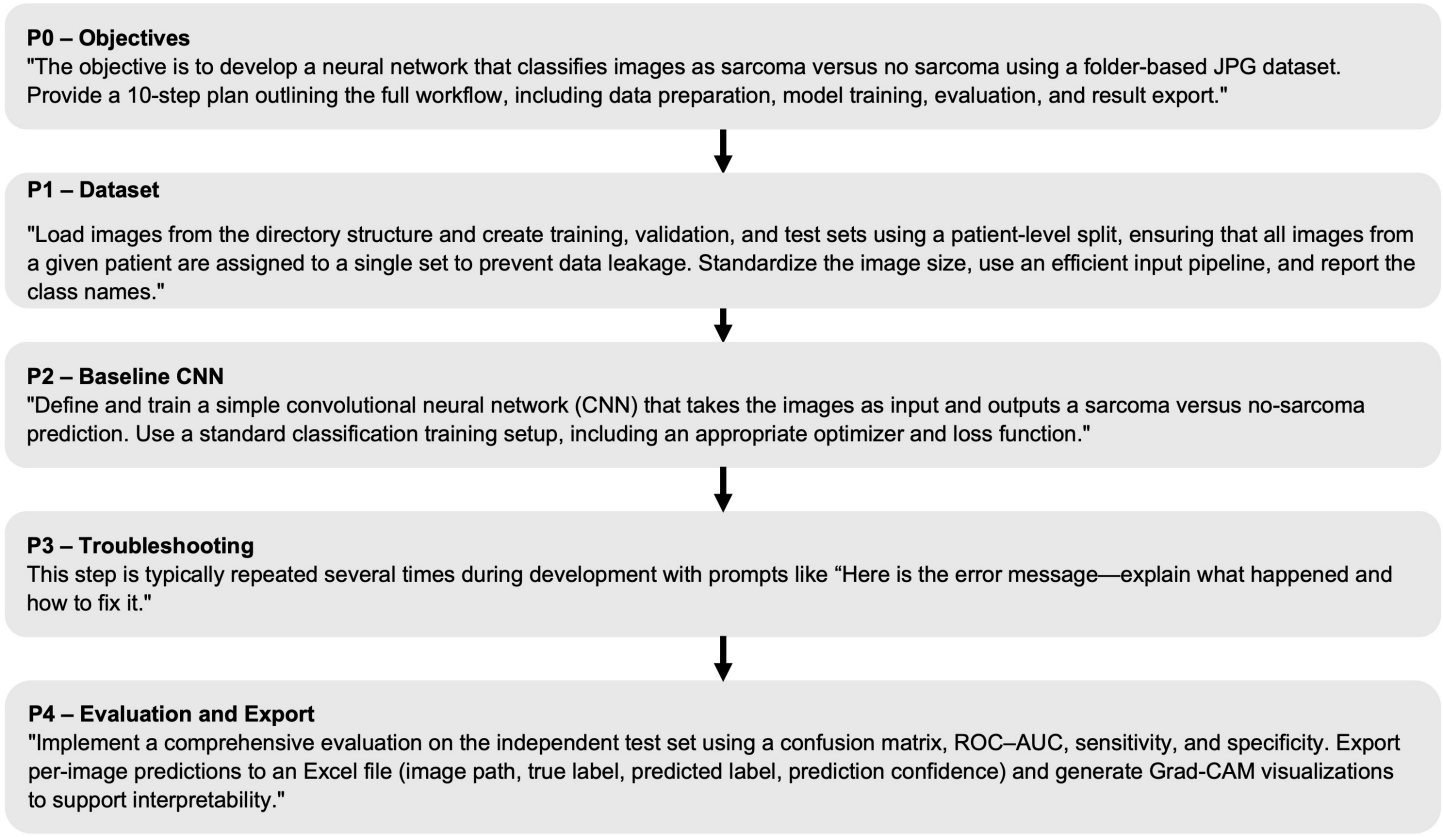
Prompt-driven development workflow (P0–P4) for CNN-based sarcoma classification. The figure summarizes the iterative stages: (P0) objective and evaluation metrics; (P1) dataset preparation (patient-level splitting, modality handling); (P2) baseline CNN definition and training configuration; (P3) iterative debugging and refinement (e.g., preprocessing consistency, class-balance checks, leakage checks); and (P4) final evaluation on the held-out test set, including result export and interpretability outputs.

ChatGPT-4.0, accessed 10-12/2023, was used only as a coding assistant (code suggestions and debugging) and did not define the scientific method, labels, or model design decisions (**Figure 3**). The generated code was verified by (i) independent code review by the authors, (ii) sanity checks on data ingestion (image counts per split, class distribution), (iii) explicit verification of patient-level split integrity (no patient overlap across subsets), (iv) reproducibility checks (fixed random seeds; repeated runs), and (v) consistency checks against reference TensorFlow/Keras documentation and expected output shapes and metrics. ChatGPT outputs were treated as non-authoritative suggestions and were accepted only after manual verification and successful execution.

### Compilation and training

**Figure 4:**
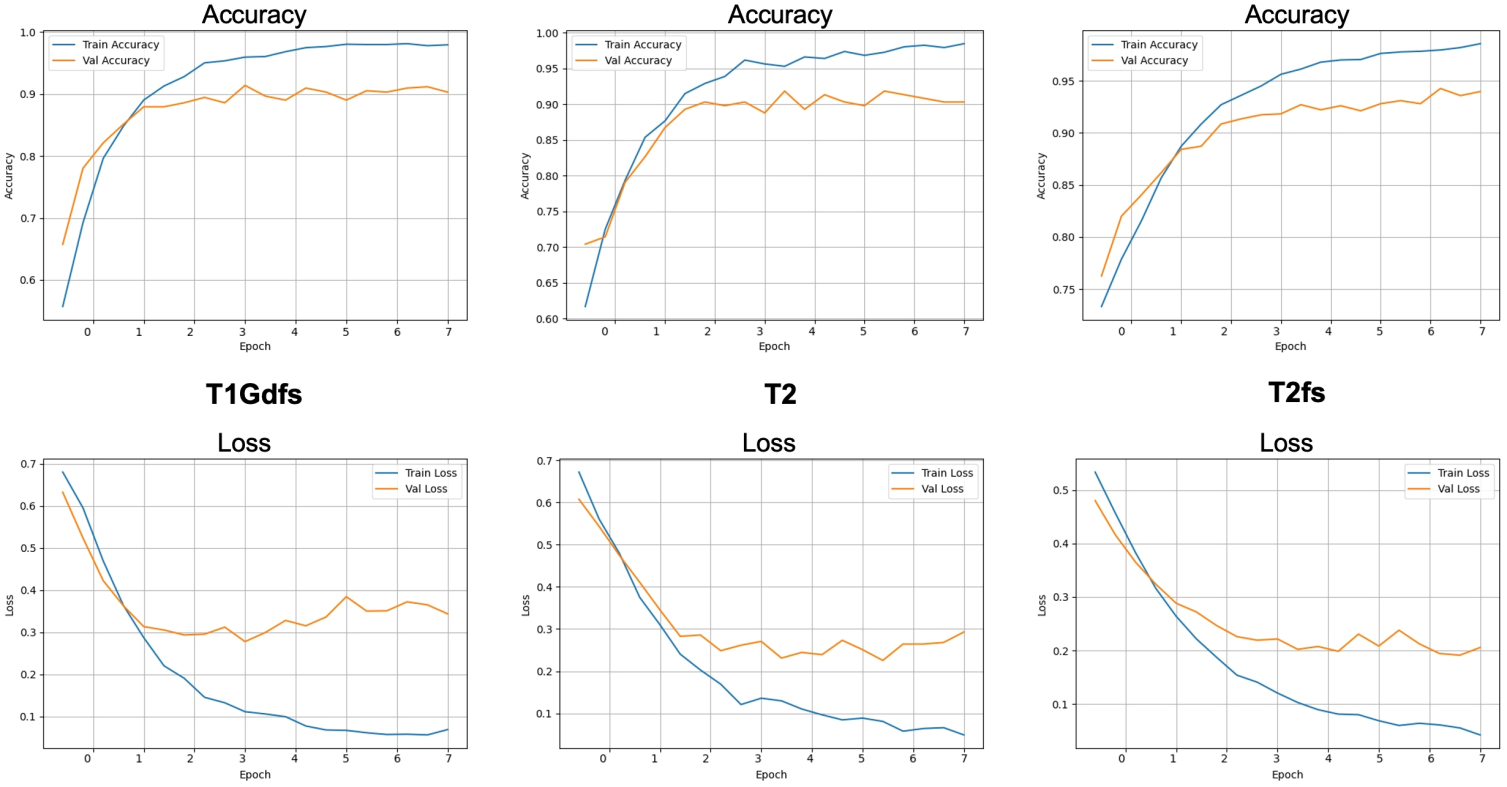
Training dynamics across representative MRI sequences (T1Gd, T2 und T2fs learning curves). Learning curves of the CNN for binary slice-level classification (tumor present vs. absent). Top row: accuracy per epoch; bottom row: loss per epoch. Training and validation curves are shown in blue and orange, respectively. Epoch labels are mapped to 0–7.

The optimizer learning rate was set to 1 × 10^−3^ (Adam). The choice of eight epochs was based on pilot experiments and inspection of learning curves: training was continued until validation loss/accuracy stabilized, and no further consistent improvement was observed within the explored range (**Figure 4**). In addition, early stopping (monitoring validation loss with patience = [X]) was implemented as a safeguard against overfitting; when enabled, the final model corresponds to the bestvalidation-loss checkpoint.

### Hyperparameter exploration and model selection

We performed an exploratory tuning phase around the initial CNN setup. This included varying commonly influential hyperparameters such as optimization settings (e.g., learning rate), batch size, and model capacity/regularization choices within a reasonable range, while monitoring validation performance and learning-curve behavior. Based on these exploratory runs, we report the best-performing configuration observed during this process (**Figure 4**). Importantly, model selection was guided by the validation set.

### Performance evaluation

Training and validation loss and accuracy were tracked across epochs (Matplotlib) to monitor convergence and learning behavior. After training, the model was evaluated on the validation set, and predictions were reviewed for correctly and incorrectly classified samples. The test set was reserved exclusively for the final performance assessment.

### Comparative analysis

To be more comparable, we standardized results after Maier-Hein et al. (21).

In addition to sensitivity, specificity, and accuracy, balanced accuracy (Sensitivity + Specificity)/2, is less affected by class imbalance), AUC (area under the ROC curve; threshold-independent measure of class separability), and F1 score (harmonic mean of precision and recall) was reported across different sequence types (T1, contrast-enhanced T1, T2, and fat-suppressed T2), as summarized in **Table 3**.

**Table 3:** Diagnostic test-set performance (patient-level split) for the CNNs. All metrics are computed exclusively on the test set for the pooled multi-modality (“Overall”) CNN and the modality-specific CNNs (T1, T1Gd, T2, T2fs).

Metric	Overall	T1	T1 Gd	T2	T2fs
Sensitivity	0.90	0.8	0.85	0.83	0.91
Specificity	0.97	0.93	0.91	0.94	0.95
Negative Predictive Value	0.95	0.91	0.86	0.87	0.97
Positive Predictive Value	0.95	0.86	0.90	0.91	0.86
Accuracy	0.95	0.89	0.88	0.89	0.94
Balanced Accuracy	0.93	0.88	0.87	0.88	0.93
AUC	0.96	0.94	0.95	0.97	0.98
F1 score	0.86	0.87	0.88	0.87	0.88

Metrics include sensitivity, specificity, negative predictive value, positive predictive value, accuracy, and balanced accuracy. AUC, Area Under the ROC Curve (Discrimination across thresholds); F1-Score: Precision–recall balance.

### Application tool development

An application tool was developed that allows users to import images in DICOM, JPG, or PNG format and obtain a binary prediction (“tumor present” vs. “tumor absent”). The output comprises the predicted class probability and a Grad-CAM heatmap for qualitative visualization of regions contributing to the model’s decision. Grad-CAM was computed from the final convolutional layer by weighting feature maps with gradients of the predicted class score and projecting the resulting relevance map back to image space as an overlay (red = higher model-attributed relevance; green/blue = lower relevance) (22).

## Results

The ChatGPT-assisted adaptation of the CNN was completed successfully and resulted in clear performance gains across the investigated MRI sequences (T1, T1Gd, T2, and T2fs) using modality-specific CNNs and an additional pooled multi-modality (“Overall”) CNN (**Figure 4**, **Table 3**). Across modalities, training converged reliably with decreasing loss and increasing accuracy, indicating that the model can learn robust tumor-related image features under heterogeneous acquisition conditions (**Figure 4**, **Table 3**).

### Performance details

- T2 (*modality-specific CNN*).The model started with a loss of 0.67 and an accuracy of 61.7%, improving to a final loss of 0.07. Validation performance increased substantially, reaching a peak validation accuracy of 91.8% (training log). On the test set, accuracy was 88.1% (**Table 3**).
- T2fs (*modality-specific CNN*). The model improved from an initial loss of 0.54 and accuracy of 76.3% to a final loss of 0.04. Test accuracy reached 93.9% (**Table 3**), indicating excellent discriminative performance in this fat-suppressed sequence.
- T1Gd (*modality-specific CNN*). Starting at a loss of 0.68 and accuracy of 65.7%, performance improved to a loss of 0.07 with a validation accuracy of 90.3%. Test accuracy was 87.3% (**Table 3**), suggesting robust but slightly lower generalization compared with T2fs.
- T1 (*modality-specific CN*N). The model started with a loss of 0.63 and accuracy of 65.9%, improving to a loss of 0.05 and a validation accuracy of 98.3%. Test accuracy was 89.4% (**Table 3**).

For the pooled multi-modality (“Overall”) CNN the model improved from a combined loss of 0.64 and accuracy of 66.3% to a test accuracy of 95.2% (**Table 3**).

Here, the “combined loss” is used as a descriptive scalar to compare optimization progress across sequences and is defined as the mean of per-modality cross-entropy losses:


$$L_{combined}=\left(1/M\right)\cdot \sum_{m=1.}^{M}L_{m}$$

Overall sensitivity and specificity of the pooled multi-modality (“Overall”) CNN were 90.1% and 97.4%, indicating consistent performance across different imaging settings.

### Learning curves and generalization behavior

The learning curves (**Figure 4**) show rapid improvements during early epochs followed by a plateau. For the T2 run, validation accuracy increased from 70.4% (epoch 1) to 91.8% (best), while validation AUC improved from 0.742 to 0.970 (training log). Notably, the best validation accuracy was reached before the final epoch, and the final validation accuracy (around 90.3%) was slightly lower than the peak, consistent with mild overfitting after the optimum epoch range.

### Visualization and interpretability

**Figure 5:**
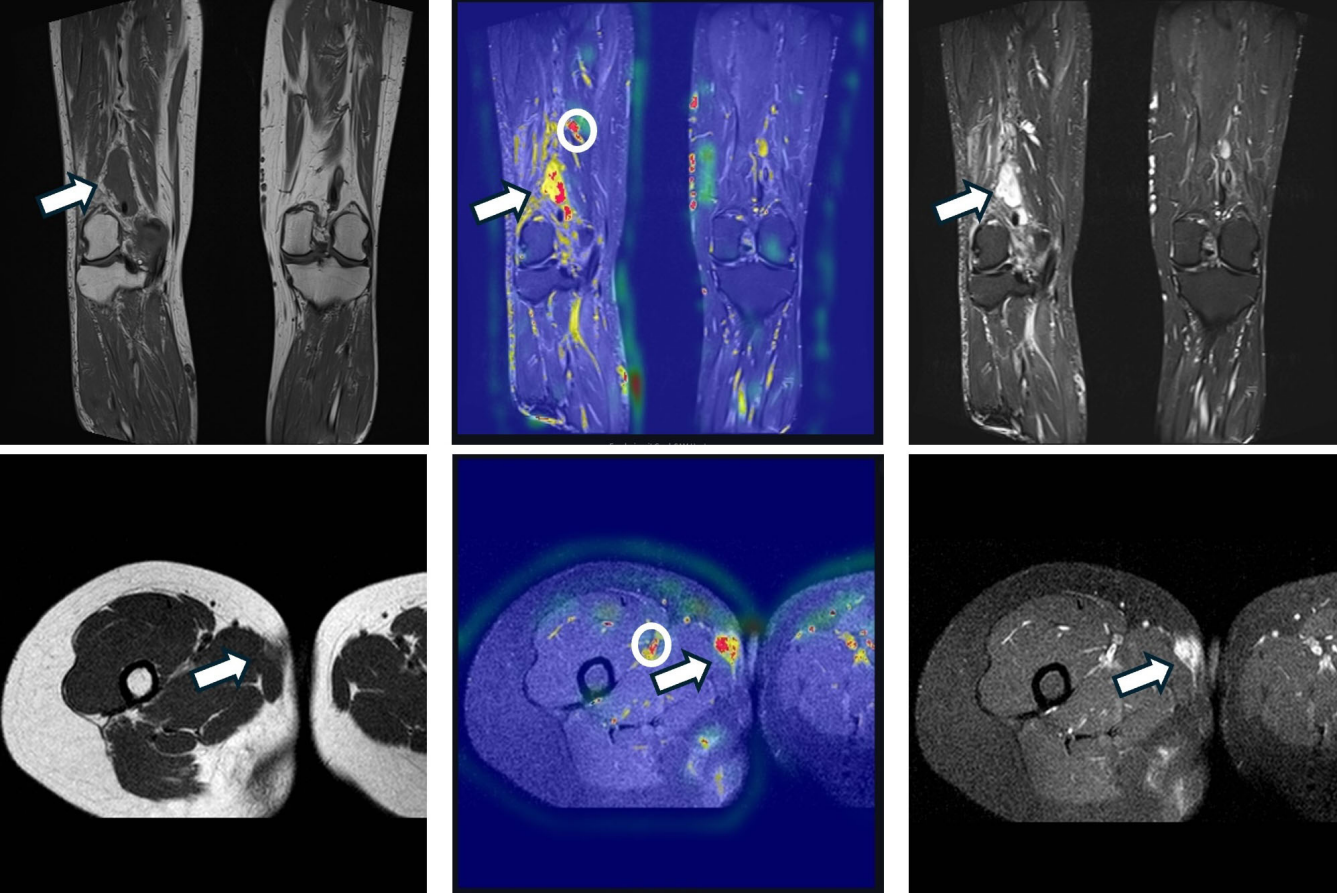
Representative MRI examples illustrating tumor localization and annotation imperfections. Top row: popliteal soft-tissue sarcoma (patient “export 8”), coronal plane—T1-weighted image (left), Grad-CAM heatmap (middle), and T2 fat-suppressed image (right). Bottom row: medial thigh sarcoma (patient “export 1”), axial plane—T1-weighted image (left), Grad-CAM heatmap (middle), and T2 fat-suppressed image (right). Arrows indicate the tumor region; circles mark areas with visible label inaccuracies.

**Figure 6:**
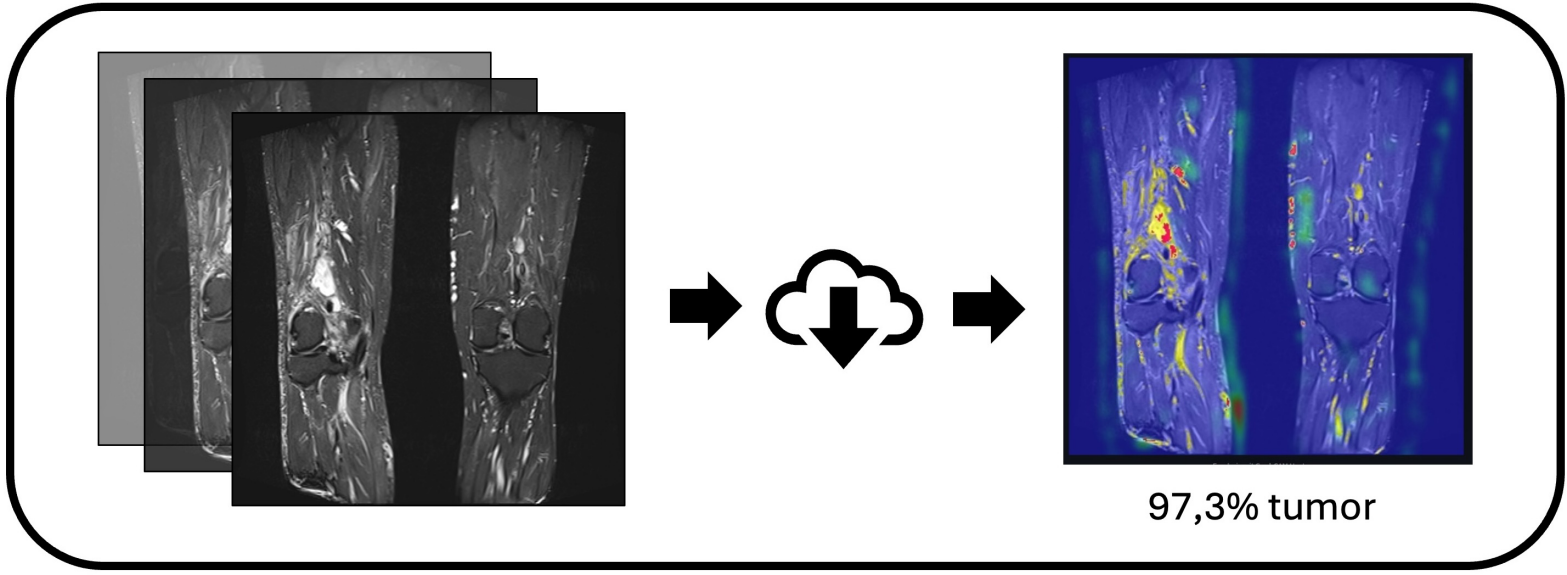
Application workflow for automated soft-tissue sarcoma detection. Schematic depiction of the developed app pipeline: image import, preprocessing, CNN inference, and output generation. The interface provides both visual feedback (e.g., highlighted regions) and probabilistic predictions (sarcoma vs. no sarcoma), supporting rapid screening and transparent result communication.

To enhance interpretability, Grad-CAM heatmaps were generated (**Figure 5**). The activations predominantly highlighted regions corresponding to tumor location; however, occasional attention in non-tumorous tissue suggests residual false-positive drivers that should be explored further (e.g., bias from edema, post-contrast enhancement patterns, or anatomical edges). For translational evaluation, a prototype application was implemented to import DICOM/JPG/PNG images via drag-and-drop (**Figure 6**).

## Discussion

This study demonstrates that an existing CNN can be adapted—using ChatGPT-assisted development—to detect soft tissue sarcoma on MRI across T1, T1Gd, T2, and T2fs (**Figure 4**, **Table 3**). We report results for modality-specific CNNs (trained separately on T1-only, T1Gd-only, T2-only, and T2fs-only data) and for an additional pooled multi-modality (“Overall”) CNN trained via pooled training across sequences. Across these experiments, training showed a consistent reduction in loss and rising accuracy, indicating stable optimization and suggesting that even a comparatively simple CNN can learn diagnostically relevant patterns in heterogeneous MRI data (23–25). The strongest gains were observed for T2fs, where performance improved markedly over the training horizon, supporting the notion that sequence-specific contrast characteristics can be highly informative for tumor detection.

The results must be interpreted cautiously due to the limited cohort size (54 patients) and the slice-based nature of the dataset. Although we applied patient-level splitting, the train/validation/test groups were not explicitly balanced (e.g., by class proportions or acquisition characteristics), which can make performance estimates sensitive to the split composition.

In addition, hyperparameter adjustments were performed using training/validation sets, while an independent held-out test set was reserved to eliminate information leakage (i.e., model-selection leakage).

Strategic randomization during training may further support generalization by reducing reliance on non-generalizable patterns (24, 26).

To mitigate the limitations of accuracy under potential class imbalance, balanced accuracy was reported in line with recommendations for medical AI evaluation (21).

High sensitivity and specificity are clinically important for minimizing missed tumors and false alarms (16, 27), but they do not fully capture stability under dataset shifts; therefore, larger datasets and external validation across diverse cohorts are essential to assess generalizability and mitigate dataset bias (28–35). Although MR images were acquired on multiple MRI scanners, external validation on independent datasets remains necessary to confirm generalizability across different acquisition settings and imaging protocols (36).

Beyond performance metrics, the broader rationale for AI support in diagnostics is well established (26, 37). In the context of regulatory expectations and the need for human oversight, decision support tools may improve efficiency and reduce error in radiology, where visual perception and uncertainty-driven decision-making intersect (38–42). For future clinical translation, the selection of appropriate radiomics features and integration into user-friendly workflows will be important (10, 11, 43–49).

Interpretability remains a key challenge for CNNs (22, 50). Grad-CAM heatmaps often highlighted plausible tumor-related regions (**Figure 5**), but occasional emphasis on irrelevant areas is consistent with known limitations of saliency methods and gradient-related artifacts—issues that can be amplified in small datasets (22, 50).

More training data and stronger priors (e.g., pretrained/foundation models) may improve robustness and localization fidelity (25, 29, 51). Finally, the present model does not distinguish soft tissue sarcoma subtypes, which limits clinical granularity. There is currently no safe AI-supported differentiation between tumor entities, although promising approaches are emerging (1, 3, 33, 52–54).

Given that AI performance can vary by task and modality (21), future work may require tailored CNNs and larger, better-controlled datasets. Practical directions include deploying simple, fast algorithms in well-designed tools as part of routine workflows to reduce bias and support general use (28, 32, 35, 55–58), followed by more advanced predictive tasks such as recurrence prediction and radio-histopathological modeling (16, 27, 59–61). Prior work suggests that, in selected settings, AI can match or exceed radiologist performance (62); combining robust model development with clinically meaningful features may further improve sensitivity/specificity and enable therapy-relevant conclusions (17, 18, 63–65), supporting imaging-based scoring and decision frameworks (30, 66–69).

Our prototype application (**Figure 6**), which supports drag-and-drop image uploads and provides both Grad-CAM visualization and tumor probability output, remains early-stage but demonstrates the feasibility of building a practical tool with relatively simple means that—after further development—could facilitate clinical workflows and everyday practice.

## Conclusion

This study successfully demonstrated the adaptability of convolutional neural networks (CNNs) in detecting soft-tissue sarcomas in extremities, showing significant improvements in diagnostic accuracy and efficiency. By customizing an existing CNN model for this purpose and integrating AI tools like ChatGPT, we enhanced model accessibility and reduced the need for specialized IT expertise. Future research is essential to fully explore the potential of AI in medical diagnostics and its practical applications in clinical settings. Overall, this study underscores the promise of AI in enhancing diagnostic processes in oncology, potentially improving patient outcomes by making advanced diagnostic tools more widely accessible.

## Data Availability

The original contributions presented in the study are included in the article/supplementary material. Further inquiries can be directed to the corresponding author.

## References

[1] Crombé A Roulleau-Dugage M Italiano A . The diagnosis, classification, and treatment of sarcoma in this era of artificial intelligence and immunotherapy. Cancer Commun (Lond). (2022) 42:1288–313. doi: 10.1002/cac2.12373, PMID: 36260064 PMC9759765

[2] Sedaghat S Sedaghat M Meschede J Jansen O Both M . Diagnostic value of MRI for detecting recurrent soft-tissue sarcoma in a long-term analysis at a multidisciplinary sarcoma center. BMC Cancer. (2021) 21:398. doi: 10.1186/s12885-021-08113-y, PMID: 33849475 PMC8042876

[3] Bourcier K Le Cesne A Tselikas L Adam J Mir O Honore C . Basic knowledge in soft tissue sarcoma. Cardiovasc Intervent Radiol. (2019) 42:1255–61. doi: 10.1007/s00270-019-02259-w, PMID: 31236647

[4] Wibmer C Leithner A Zielonke N Sperl M Windhager R . Increasing incidence rates of soft tissue sarcomas? A population-based epidemiologic study and literature review. Ann Oncol. (2010) 21:1106–11. doi: 10.1093/annonc/mdp415, PMID: 19858086

[5] Pizzato M Collatuzzo G Santucci C Malvezzi M Boffetta P Comandone A . Mortality patterns of soft-tissue sarcomas worldwide up to 2018, with predictions for 2025. Eur J Cancer Prev. (2023) 32:71–80. doi: 10.1097/CEJ.0000000000000768, PMID: 36346699

[6] Weiss MC . Systemic treatment of soft tissue sarcomas in the geriatric population. Curr Treat Options Oncol. (2022) 23:855–63. doi: 10.1007/s11864-022-00972-2, PMID: 35389146

[7] Arunachalam P Venkatakrishnan P Janakiraman N . ” Histopathology image classification for soft tissue sarcoma in limbs using artificial neural networks“ In: 2021 6th international conference on inventive computation technologies (ICICT). Piscataway, NJ, USA: IEEE. (2021) pp. 778–85.

[8] Peeken JC Goldberg T Knie C Komboz B Bernhofer M Pasa F . Treatment-related features improve machine learning prediction of prognosis in soft tissue sarcoma patients. Strahlentherapie und Onkologie. (2018) 194(9):824–34. doi: 10.1007/s00066-018-1294-2, PMID: 29557486

[9] Sedaghat S Schmitz F Grözinger M Sedaghat M . Malignant peripheral nerve sheath tumours in magnetic resonance imaging: primary and recurrent tumour appearance, post-treatment changes, and metastases. Pol J Radiol. (2020) 85:e196–201. doi: 10.5114/pjr.2020.94687, PMID: 32419885 PMC7218449

[10] Boudabbous S Hamard M Saiji E Gorican K Poletti PA Becker M . What morphological MRI features enable differentiation of low-grade from high-grade soft tissue sarcoma? BJR Open. (2022) 4:20210081. doi: 10.1259/bjro.20210081, PMID: 36105415 PMC9459866

[11] Sedaghat S Salehi Ravesh M Sedaghat M Both M Jansen O . Configuration of soft-tissue sarcoma on MRI correlates with grade of Malignancy. Radiol Oncol. (2021) 55:158–63. doi: 10.2478/raon-2021-0007, PMID: 33600679 PMC8042815

[12] Crombé A Marcellin PJ Buy X Stoeckle E Brouste V Italiano A . Soft-tissue sarcomas: assessment of MRI features correlating with histologic grade and patient outcome. Radiology. (2019) 291:710–21. doi: 10.1148/radiol.2019181659, PMID: 30964422

[13] Chhabra A Ashikyan O Slepicka C Dettori N Hwang H Callan A . Conventional MR and diffusion-weighted imaging of musculoskeletal soft tissue Malignancy: correlation with histologic grading. Eur Radiol. (2019) 29:4485–94. doi: 10.1007/s00330-018-5845-9, PMID: 30511176

[14] Gatta G van Der Zwan JM Casali PG Siesling S Dei Tos AP Kunkler I . Rare cancers are not so rare: the rare cancer burden in Europe. Eur J Cancer. (2011) 47:2493–511. doi: 10.1016/j.ejca.2011.08.008, PMID: 22033323

[15] Greenlee RT Goodman MT Lynch CF Platz CE Havener LA Howe HL . The occurrence of rare cancers in U.S. adults, 1995-2004. Public Health Rep. (2010) 125:28–43. doi: 10.1177/003335491012500106, PMID: 20402194 PMC2789814

[16] Gitto S Interlenghi M Cuocolo R Salvatore C Giannetta V Badalyan J . MRI radiomics-based machine learning for classification of deep-seated lipoma and atypical lipomatous tumor of the extremities. Radiol Med. (2023) 128:989–98. doi: 10.1007/s11547-023-01657-y, PMID: 37335422 PMC10338387

[17] Escobar T Vauclin S Orlhac F Nioche C Pineau P Champion L . Voxel-wise supervised analysis of tumors with multimodal engineered features to highlight interpretable biological patterns. Med Phys. (2022) 49:3816–29. doi: 10.1002/mp.15603, PMID: 35302238 PMC9325536

[18] Gao Y Ghodrati V Kalbasi A Fu J Ruan D Cao M . Prediction of soft tissue sarcoma response to radiotherapy using longitudinal diffusion MRI and a deep neural network with generative adversarial network-based data augmentation. Med Phys. (2021) 48:3262–372. doi: 10.1002/mp.14897, PMID: 33908045

[19] Ronneberger O Fischer P Brox T . U-net: Convolutional networks for biomedical image segmentation, in: Medical image computing and computer-assisted intervention–MICCAI 2015: 18th international conference *Munich, Germany, October 5-9, 2015. Cham, Switzerland proceedings, part III 18 pp*. Cham, Switzerland: Springer (2015) 234–41.

[20] TensorFlow . Load and preprocess images. Mountain View, CA, USA: TensorFlow (2022). Available online at: https://www.tensorflow.org/tutorials/load_data/images (Accessed March 1, 2024).

[21] Maier-Hein L Reinke A Godau P Tizabi MD Buettner F Christodoulou E . Metrics reloaded: recommendations for image analysis validation. Nat Methods. (2024) 21:195–212. doi: 10.1038/s41592-023-02151-z, PMID: 38347141 PMC11182665

[22] Selvaraju RR Cogswell M Das A Vedantam R Parikh D Batra D . Grad-CAM: visual explanations from deep networks via gradient-based localization. Int J Comput Vision. (2020) 128:336–59. doi: 10.1007/s11263-019-01228-7, PMID: 41810330

[23] Hosny A Parmar C Quackenbush J Schwartz LH Aerts H . Artificial intelligence in radiology. Nat Rev Cancer. (2018) 18:500–10. doi: 10.1038/s41568-018-0016-5, PMID: 29777175 PMC6268174

[24] Al-Obeidat F Rashid A Hafez W Gibbaoui H Ayoub G Al Ameer S . The accuracy of artificial intelligence in the diagnosis of soft tissue sarcoma: A systematic review and meta-analysis. Curr Probl Surg. (2025) 66:101743. doi: 10.1016/j.cpsurg.2025.101743, PMID: 40306879

[25] Tölle M Garthe P Scherer C Seliger JM Leha A Krüger N . Federated foundation model for cardiac CT imaging. arXiv. (2024). doi: 10.48550/arXiv.2407.07557, PMID: 41363103

[26] Zendel O Murschitz M Humenberger M Herzner W . How good is my test data? Introducing safety analysis for computer vision. Int J Comput Vision. (2017) 125:95–109. doi: 10.1007/s11263-017-1020-z, PMID: 41810330

[27] Dai M Liu Y Hu Y Li G Zhang J Xiao Z . Combining multiparametric MRI features-based transfer learning and clinical parameters: application of machine learning for the differentiation of uterine sarcomas from atypical leiomyomas. Eur Radiol. (2022) 32:7988–97. doi: 10.1007/s00330-022-08783-7, PMID: 35583712

[28] Ansart M Epelbaum S Bassignana G Bône A Bottani S Cattai T . Predicting the progression of mild cognitive impairment using machine learning: A systematic, quantitative and critical review. Med Image Anal. (2021) 67:101848. doi: 10.1016/j.media.2020.101848, PMID: 33091740

[29] Moor M Banerjee O Abad ZSH Krumholz HM Leskovec J Topol EJ . Foundation models for generalist medical artificial intelligence. Nature. (2023) 616:259–65. doi: 10.1038/s41586-023-05881-4, PMID: 37045921

[30] Peeken JC Asadpour R Specht K Chen EY Klymenko O Akinkuoroye V . MRI-based delta-radiomics predicts pathologic complete response in high-grade soft-tissue sarcoma patients treated with neoadjuvant therapy. Radiother Oncol. (2021) 164:73–82. doi: 10.1016/j.radonc.2021.08.023, PMID: 34506832

[31] Roberts M Driggs D Thorpe M Gilbey J Yeung M Ursprung S . Common pitfalls and recommendations for using machine learning to detect and prognosticate for COVID-19 using chest radiographs and CT scans. Nat Mach Intell. (2021) 3:199–217. doi: 10.1038/s42256-021-00307-0, PMID: 41803196

[32] Sedaghat S . Success through simplicity: what other artificial intelligence applications in medicine should learn from history and chatGPT. Ann BioMed Eng. (2023) 51:2657–8. doi: 10.1007/s10439-023-03287-x, PMID: 37332004 PMC10632240

[33] Sedaghat S Salehi Ravesh M Sedaghat M Meschede J Jansen O Both M . Does the primary soft-tissue sarcoma configuration predict configuration of recurrent tumors on magnetic resonance imaging? Acta Radiol. (2022) 63:642–51. doi: 10.1177/02841851211008381, PMID: 33853376

[34] Sedaghat S Schmitz F Krieger A Sedaghat M Reichardt B . Appearance of recurrent adult fibrosarcoma of the soft tissue and loco-regional post-treatment changes on MRI follow-up. Eur J Plast Surg. (2021) 44:97–102. doi: 10.1007/s00238-020-01669-1, PMID: 41810330

[35] Varoquaux G Cheplygina V . Machine learning for medical imaging: methodological failures and recommendations for the future. NPJ Digit Med. (2022) 5:48. doi: 10.1038/s41746-022-00592-y, PMID: 35413988 PMC9005663

[36] Yu AC Mohajer B Eng J . External validation of deep learning algorithms for radiologic diagnosis: A systematic review. Radiol Artif Intell. (2022) 4:e210064. doi: 10.1148/ryai.210064, PMID: 35652114 PMC9152694

[37] Lodwick GS . Computer-aided diagnosis in radiology: A research plan. Invest Radiol. (1966) 1:72–80. doi: 10.1097/00004424-196601000-00032, PMID: 5910559

[38] Haselmann V Schoenberg SO Neumaier M Froelich MF . Integrated diagnostics. Die Radiologie. (2022) 62:11–6. doi: 10.1007/s00117-022-01043-1, PMID: 35819468

[39] Noy S Zhang W . Experimental evidence on the productivity effects of generative artificial intelligence. Science. (2023) 381:187–92. doi: 10.1126/science.adh2586, PMID: 37440646

[40] Boland GW Guimaraes AS Mueller PR . The radiologist’s conundrum: benefits and costs of increasing CT capacity and utilization. Eur Radiol. (2009) 19:9–11; discussion 12. doi: 10.1007/s00330-008-1159-7, PMID: 18766347

[41] McDonald RJ Schwartz KM Eckel LJ Diehn FE Hunt CH Bartholmai BJ . The effects of changes in utilization and technological advancements of cross-sectional imaging on radiologist workload. Acad Radiol. (2015) 22:1191–8. doi: 10.1016/j.acra.2015.05.007, PMID: 26210525

[42] Fitzgerald R . Error in radiology. Clin Radiol. (2001) 56:938–46. doi: 10.1053/crad.2001.0858, PMID: 11795921

[43] Sculley D Holt G Golovin D Davydov E Phillips T Ebner D . Hidden technical debt in machine learning systems. Adv Neural Inf Process Syst. (2015) 28:2503–11. doi: 10.48550/arXiv.2310.02207, PMID: 41363103

[44] Zhu N Meng X Wang Z Hu Y Zhao T Fan H . Radiomics in diagnosis, grading, and treatment response assessment of soft tissue sarcomas: A systematic review and meta-analysis. Acad Radiol. (2024) 31:3982–92. doi: 10.1016/j.acra.2024.03.029, PMID: 38772802

[45] Schmitz F Sedaghat S . Inferring Malignancy grade of soft tissue sarcomas from magnetic resonance imaging features: A systematic review. Eur J Radiol. (2024) 177:111548. doi: 10.1016/j.ejrad.2024.111548, PMID: 38852328

[46] Löwenthal D Zeile M Niederhagen M Fehlberg S Schnapauff D Pink D . Differentiation of myxoid liposarcoma by magnetic resonance imaging: a histopathologic correlation. Acta Radiol. (2014) 55:952–60. doi: 10.1177/0284185113508114, PMID: 24123962

[47] Kelly BS Judge C Bollard SM Clifford SM Healy GM Aziz A . Radiology artificial intelligence: a systematic review and evaluation of methods (RAISE). Eur Radiol. (2022) 32:7998–8007. doi: 10.1007/s00330-022-08784-6, PMID: 35420305 PMC9668941

[48] Pfaehler E Zhovannik I Wei L Boellaard R Dekker A Monshouwer R . A systematic review and quality of reporting checklist for repeatability and reproducibility of radiomic features. Phys Imaging Radiat Oncol. (2021) 20:69–75. doi: 10.1016/j.phro.2021.10.007, PMID: 34816024 PMC8591412

[49] Schmitz F Voigtländer H Jang H Schlemmer HP Kauczor HU Sedaghat S . Predicting the Malignancy grade of soft tissue sarcomas on MRI using conventional image reading and radiomics. Diagnostics (Basel). (2024) 14:2220. doi: 10.3390/diagnostics14192220, PMID: 39410624 PMC11482587

[50] Reyes M Meier R Pereira S Silva CA Dahlweid FM von Tengg-Kobligk H . On the interpretability of artificial intelligence in radiology: challenges and opportunities. Radiology: Artif Intell. (2020) 2:e190043. doi: 10.1148/ryai.2020190043, PMID: 32510054 PMC7259808

[51] Gurnee W Tegmark M . Language models represent space and time. arXiv. (2023). doi: 10.48550/arXiv.2310.02207, PMID: 41363103

[52] Sedaghat S Schmitz F Sedaghat M Nicolas V . Appearance of recurrent dermatofibrosarcoma protuberans in postoperative MRI follow-up. J Plast Reconstr Aesthet Surg. (2020) 73:1960–5. doi: 10.1016/j.bjps.2020.08.089, PMID: 32952057

[53] Sedaghat S Sedaghat M Krohn S Jansen O Freund K Streitbürger A . Long-term diagnostic value of MRI in detecting recurrent aggressive fibromatosis at two multidisciplinary sarcoma centers. Eur J Radiol. (2021) 134:109406. doi: 10.1016/j.ejrad.2020.109406, PMID: 33254066

[54] Blackledge MD Winfield JM Miah A Strauss D Thway K Morgan VA . Supervised machine-learning enables segmentation and evaluation of heterogeneous post-treatment changes in multi-parametric MRI of soft-tissue sarcoma. Front Oncol. (2019) 9:941. doi: 10.3389/fonc.2019.00941, PMID: 31649872 PMC6795696

[55] Hermessi H Mourali O Zagrouba E . ” Transfer learning with multiple convolutional neural networks for soft tissue sarcoma MRI classification“ In: Eleventh International Conference on Machine Vision (ICMV 2018), Bellingham, WA, USA: SPIE. (2019) pp. 706–12.

[56] Topol EJ . High-performance medicine: the convergence of human and artificial intelligence. Nat Med. (2019) 25:44–56. doi: 10.1038/s41591-018-0300-7, PMID: 30617339

[57] Sendak MP D’Arcy J Kashyap S Gao M Nichols M Corey K . A path for translation of machine learning products into healthcare delivery. EMJ Innov. (2020) 10:19–00172. doi: 10.33590/emjinnov/19-00172

[58] Torralba A Efros AA . (2011). ” Unbiased look at dataset bias“, In: CVPR 2011, Bellingham, WA, USA: IEEE. pp. 1521–8.

[59] Xu W Hao D Hou F Zhang D Wang H . Soft tissue sarcoma: preoperative MRI-based radiomics and machine learning may be accurate predictors of histopathologic grade. AJR Am J Roentgenol. (2020) 215:963–9. doi: 10.2214/ajr.19.22147, PMID: 32755226

[60] Cay N Mendi BAR Batur H Erdogan F . Discrimination of lipoma from atypical lipomatous tumor/well-differentiated liposarcoma using magnetic resonance imaging radiomics combined with machine learning. Jpn J Radiol. (2022) 40:951–60. doi: 10.1007/s11604-022-01278-x, PMID: 35430677

[61] Roller LA Wan Q Liu X Qin L Chapel D Burk KS . MRI, clinical, and radiomic models for differentiation of uterine leiomyosarcoma and leiomyoma. Abdom Radiol (NY). (2024) 49:1522–33. doi: 10.1007/s00261-024-04198-8, PMID: 38467853

[62] Malinauskaite I Hofmeister J Burgermeister S Neroladaki A Hamard M Montet X . Radiomics and machine learning differentiate soft-tissue lipoma and liposarcoma better than musculoskeletal radiologists. Sarcoma. (2020) 2020:7163453. doi: 10.1155/2020/7163453, PMID: 31997918 PMC6969992

[63] Foersch S Eckstein M Wagner DC Gach F Woerl AC Geiger J . Deep learning for diagnosis and survival prediction in soft tissue sarcoma. Ann Oncol. (2021) 32:1178–87. doi: 10.1016/j.annonc.2021.06.007, PMID: 34139273

[64] Holbrook MD Blocker SJ Mowery YM Badea A Qi Y Xu ES . MRI-based deep learning segmentation and radiomics of sarcoma in mice. Tomography. (2020) 6:23–33. doi: 10.18383/j.tom.2019.00021, PMID: 32280747 PMC7138523

[65] Kim J McIlwain S Lee I Ong I Cho S . Prognostic value of baseline FDG PET/CT and MR images using machine learning algorithm in soft tissue sarcoma. Soc Nucl Med. (2018) 59:129.

[66] Li RH Zhou Q Li AB Zhang HZ Lin ZQ . A nomogram to predict metastasis of soft tissue sarcoma of the extremities. Med (Baltimore). (2020) 99:e20165. doi: 10.1097/md.0000000000020165, PMID: 32481285 PMC7250057

[67] Spraker MB Wootton L Hippe DS Chaovalitwongse W Macomber M Chapman T . Radiomic signature extracted from magnetic resonance imaging predicts outcomes in soft tissue sarcoma. Int J Radiat Oncology Biology Phys. (2017) 99:S78–9. doi: 10.1016/j.ijrobp.2017.06.190, PMID: 41810140

[68] Maussion C Coindre JM Blay JY Schutte K Camara A Le Loarer F . Multimodal prediction of metastatic relapse using federated deep learning outperforms state-of-the-art methods in soft-tissue sarcoma. Cancer Res. (2022) 82:1939–9. doi: 10.1158/1538-7445.AM2022-1939, PMID: 41120524 PMC12540783

[69] Wang H Chen H Duan S Hao D Liu J . Radiomics and machine learning with multiparametric preoperative MRI may accurately predict the histopathological grades of soft tissue sarcomas. J Magn Reson Imaging. (2020) 51:791–7. doi: 10.1002/jmri.26901, PMID: 31486565

